# Detection of *Salmonella* enteritidis, typhi and typhimurium in foods by multiplex PCR in children hospital

**DOI:** 10.1186/1471-2334-12-S1-P95

**Published:** 2012-05-04

**Authors:** Shima Nosraty, Fatemeh Fallah, Azar Sabokbar, Mehrooz Dezfoolyan, Saadat Adabian, Narges Esmaeilnejad

**Affiliations:** 1 Karaj Branch, Islamic Azad University, Karaj, Iran; 2 Pediatric Infectious Research Center, Shahid Beheshti Medical University, Tehran, Iran

## Background

Food-borne illnesses have high prevalence in the world, which are caused by consumption of water or food contaminated. Nowadays, involvement caused by Salmonella bacterium, serotype enteritidis and typhimurium are the major infection all around the world. So the purpose of this study is detection of *Salmonella* enteritidis, typhi and typhimurium in different kinds of foods by multiplex PCR method.

## Methods

The study was conducted on 170 samples of food products including, milk, beef, poultry, salad dressing and 80 samples in contact with food items such as knives, cutting boards and the hands of personnel working in a hospital’s kitchen. After collecting the samples, the standard diagnostic method for detection of bacteria were performed and after extracting the DNA, the multiplex PCR method has been used for determination of Salmonella serotypes.

## Results

From all of the tested food samples (170), 1.7% of Salmonella contamination has observed that 1.1% related to typhimurium and 0.6% related to *Salmonella* enteritidis that had separated from beef, and during the tests conducted on samples that were in contact with food items, Salmonella contamination was not detected.

## Conclusion

Results indicated that**,** quality control of food products in processing and production stages observing hygiene issues are critically important in preventing food-borne diseases. And also the methods like molecular diagnostic such as multiplex PCR besides the cultivation of bacteria and other microbes can be helpful in diagnostic confirmation.

